# A sensitive synthetic reporter for visualizing cytokinin signaling output in rice

**DOI:** 10.1186/s13007-017-0232-0

**Published:** 2017-10-27

**Authors:** Jinyuan Tao, Huwei Sun, Pengyuan Gu, Zhihao Liang, Xinni Chen, Jiajing Lou, Guohua Xu, Yali Zhang

**Affiliations:** State Key Laboratory of Crop Genetics and Germplasm Enhancement, Key Laboratory of Plant Nutrition and Fertilization in Low-Middle Reaches of the Yangtze River, Ministry of Agriculture, Nanjing Agricultural University, Weigang1, Xuanwu District, Nanjing, 210095 China

**Keywords:** Cytokinin signaling, Rice, Synthetic sensor, Two component system

## Abstract

**Background:**

Cytokinins play many essential roles in plant growth and development, mainly through signal transduction pathways. Although the cytokinin signaling pathway in rice has been clarified, no synthetic reporter for cytokinin signaling output has been reported for rice. The sensitive synthetic reporter two-component signaling sensor (*TCSn*) is used in the model plant Arabidopsis; however, whether the reporter reflects the cytokinin signaling output pattern in rice remains unclear.

**Results:**

Early-cytokinin-responsive type-A OsRR-binding element (A/G)GAT(C/T) was more clustered in the 15 type-A OsRRs than in the 13 control genes. Quantitative polymerase chain reaction analysis showed that the relative expression of seven type-A *OsRRs* in roots and shoots was significantly induced by exogenous cytokinin application, and that of seven *OsRRs*, mainly in roots, was inhibited by exogenous auxin application. We constructed a transgenic rice plant harboring a beta-glucuronidase (GUS) driven by the synthetic promoter *TCSn*. *TCSn::GUS* was expressed in the meristem of germinated rice seed and rice seedlings. Furthermore, *TCSn::GUS* expression in rice seedlings was induced specifically by exogenous cytokinin application and decreased by exogenous auxin application. Moreover, no obvious reduction in GUS levels was observed after three generations of selfing of transgenic plants, indicating that *TCSn::GUS* is not subject to transgene silencing.

**Conclusions:**

We report here a robust and sensitive synthetic sensor for monitoring the transcriptional output of the cytokinin signaling network in rice.

**Electronic supplementary material:**

The online version of this article (doi:10.1186/s13007-017-0232-0) contains supplementary material, which is available to authorized users.

## Background

The plant phytohormone cytokinin is a key growth regulator involved in regulating a wide range of developmental processes in diverse contexts throughout the plant life cycle [1–4]. Cytokinin regulates cell division mainly through histidyl–aspartyl phosphorelay signal transduction (also known as two-component systems), which consist of a histidine protein kinase that senses the input and a response regulator that mediates the output-control signal transduction pathways in many prokaryotes and in some eukaryotes [5–8]. The Arabidopsis cytokinin signal transduction pathway consists of four principal steps: histidine protein kinase (HK) sensing and signaling; transfer of the phosphoryl group to histidine phosphotransfer protein (HP); response regulator (RR)-dependent transcription activation; and a negative feedback loop through cytokinin-inducible RR genes [4, 6, 9–16].

Although the cytokinin multistep phosphorelay signal transduction pathway is present in Arabidopsis, the identification and functional characterization of the diverse signaling locales is challenging. The distribution of active cytokinin ligands in plants is difficult to determine, mainly due to the synthesis of the cytokinins involved in the complex enzymatic biosynthetic pathways in different cellular compartments [17] and the low level of precise localization (about 100-fold lower than auxin levels) with derived metabolites and different forms [18–20]. Cytokinin signaling output at the transcriptional level in Arabidopsis has been visualized using a synthetic reporter, revealing the sites of cytokinin action during wildtype development [21, 22]. Nuclear type-B RRs mediate transcriptional activation in response to phosphorelay signaling activity, whereas type-A RRs repress signaling in a negative-feedback loop. The DNA-binding domains of diverse type-B RR family members are conserved and bind a common DNA-target sequence (A/G)GAT(T/C) in vitro [23–25]. Accordingly, the concatemerized type-B ARR-binding motifs are core components of the synthetic reporter [21, 22].

Rice (*Oryza sativa* L.) is not only a major staple food worldwide but also an important model plant for monocot species research because of its small genome and the availability of its complete genome sequence [26, 27]. The cytokinin signaling pathway in rice has been clarified; however, no synthetic reporter for cytokinin signaling output in rice has been reported. The sensitive synthetic reporter two-component signaling sensor (*TCSn*) is used in the model plant Arabidopsis; however, whether the reporter reflects the cytokinin signaling output pattern in rice remains unclear. In this study, we analyzed the DNA-binding domains (5′-(A/G)GAT(T/C)-3′) in the promoters of type-A RR family members and examined the changes in the relative expression of type-A RR genes in response to phytohormones. The stronger version of *TCSn::GUS* was fused into rice plants through genetic transformation. We investigated GUS expression in different generations of transgenic rice seedlings, including in response to exogenous application of six cytokinin fractions and three other phytohormones. We report here a robust and sensitive synthetic sensor for monitoring the transcriptional output of the cytokinin signaling network in rice.

## Results and discussion

### Amino acid alignment of the Myb-like domains from type-B response regulators

We first selected and analyzed 10 rice type-B positive regulators from the phylogenetic tree described by Tsai et al. [28]. As expected (Additional file 1), the amino acid residues of the C-terminal Myb-like domain [SH(A/L)QKY(R/F)] responsible for their DNA-binding specificity are conserved between rice and Arabidopsis, which is consistent with online registration of a conserved Myb-like motif (accession number: TIGR01557, http://www.jcvi.org/cms/home/). Also, the differences in the sequences of the Myb-like domains may result in regulation of different downstream gene sets in different species.

### Analysis of the DNA-binding motif in type-A OsRRs

Cytokinins are classic plant hormones that orchestrate plant growth and physiology and affect gene expression in target cells by activating a multistep phosphorelay network. Type-B RRs, acting as transcriptional activators, mediate the final step in the signaling cascade; simultaneously, type-A RRs are immediate–early target genes of activated type-B RR proteins, which establishes a negative feedback loop to the signaling pathway [4, 15, 16, 19, 22, 29].

To identify the rice type-B RR regulator binding motif (5′-(A/G)GAT(C/T)T-3′) in rice, sharing the conserved pathway with Arabidopsis [30–32], the sequence including the promoter and part of the transcribed sequence of rice type-A *RR* genes (*OsRR1*-*15*) was analyzed (Additional file 2) [33]. Additionally, 13 genes that showed stable expression and had no relationship with stress, developmental, pharmacological, or physiological treatments [34–36], were randomly selected as negative controls. The total length of the 13 control genes (69.7 kb) was similar to that of the 15 rice type-A *RR* genes (66.9 kb).

We analyzed the frequency of motifs based on total sequence length and gene sequence. In addition to 66.9 kb from the 15 type-A OsRRs and 69.7 kb from the 13 control genes, a 66.9-kb sequence was randomly selected from the NCBI GenBank database for use as negative control sequences (Additional file 2). The motif number, distance between neighboring motifs, and relative orientation of motifs are important for type-B regulator binding. Based on the distance, motifs were separated into two groups: one with a > 6–30-bp distance and another with a 6–30-bp distance. No difference in the total number of motifs, the motif density, or the number of motifs of distance > 6–30 bp was recorded in the sequences of the 15 type-A genes, 13 control genes, and random sequences (P > 0.05); however, a significant difference was detected in the number of motifs with a 6–30-bp distance irrespective of their direction (P < 0.05). Because motif distance is important for type-B regulator binding [22], our data support the notion that the motif functions in transcription factor binding in natural promoters in rice as well as Arabidopsis. Similarly, a markedly higher mean number of motifs with a 6–30-bp distance was observed in the 15 type-A OsRRs than in the 13 control genes. No apparent bias toward tandem or inverse orientation was recorded in rice (Additional file 3), as in Arabidopsis [22]. To summarize, higher clustered motif were more in rice cytokinin signaling targets than in control genes, which supports the functionality of *TCSn* in rice.

### Changes in the relative expression of type-A *OsRRs* in shoots and roots in response to cytokinin

**Fig. 1:**
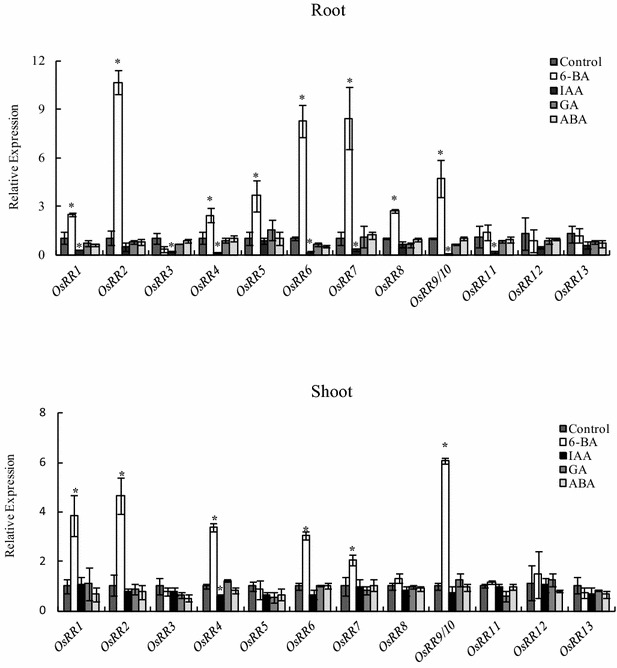
Relative expression of type-A *OsRR* family genes in response to exogenous phytohormones. Analysis was performed on wild-type plants germinated and grown hydroponically for 14 days, followed by 3 h treatment with 6-benzylaminopurine (6-BA, 100 nM), indole-3-acetic acid (IAA, 1 μM), gibberellic acid (GA, 10 μM), and abscisic acid (ABA, 10 μM). Relative mRNA levels were normalized to those of *OsACT*. Values are mean ± SD of four biological replicates. *P < 0.05 (ANOVA) between the control and the indicated treatments

Cytokinin-dependent induction of type-A RRs is, in part, dependent on transcriptional regulation by type-B RRs [9, 37]. Similar to Arabidopsis [13], the transcripts of seven rice type-A *OsRR* genes accumulated rapidly both in roots and shoots after 6-BA treatment for 3 h. Among the type-A *OsRR* genes in rice, *OsRR9* and *OsRR10* are difficult to distinguish because of their > 99% nucleotide sequence similarity [38]. Of the other six type-A *OsRR* genes, the expression of *OsRR14*/*15* was too weak to be detected in rice roots and shoots; and *OsRR3,11*-*13* transcript levels did not respond to 6-BA application both in roots and shoots. To evaluate the responses to other phytohormones of the type-A *OsRR* genes, we also assessed their expression upon exposure of rice plants to exogenous auxin (IAA), gibberellic acid (GA), and abscisic acid (ABA). Interestingly, transcripts of type-A *OsRR*s responded to exogenous IAA but not GA and ABA. Application of IAA markedly reduced the transcript levels of most type-A *OsRR* genes in rice roots, with the exception of *OsRR5, OsRR8,* and *OsRR12*-*13* (Fig. 1). This is consistent with the notion that auxin antagonizes cytokinin output by direct transcriptional activation of feedback repressors of cytokinin signaling [21, 39]. However, most genes in the shoot, the exception being *OsRR4*, did not respond to IAA application, suggesting that *OsRR* family genes in shoots and roots respond differently to exogenous auxin application.

Furthermore, *OsRR6* and *OsRR9/10* levels were higher both in shoots and roots in the control group (Additional file 4). Thus, *OsRR6* and *OsRR9/10* were selected as marker genes in subsequent experiments.

### *TCSn::GUS* expression patterns are consistent with known cytokinin functions in rice seedlings

To generate a universal cytokinin reporter, a synthetic reporter harboring the concatemerized type-B RR-binding motifs has been tested and optimized in vivo [21]. However, this system has the following limitations: TCS-induced expression is weak in certain developmental contexts in which cytokinin signaling is involved, such as in the shoot and in the vasculature. Furthermore, GFP expression in the root meristem of the seedling decreases as the number of generations increases. Accordingly, an improved version of the reporter, *TCS new* (*TCSn*), which, compared with *TCS*, is more sensitive to phosphorelay signaling in Arabidopsis and maize cellular assays while retaining its specificity, has been developed. *TCSn* includes variations in the number of binding sites, phasing, and identity of flanking nucleotides [22].

**Fig. 2:**
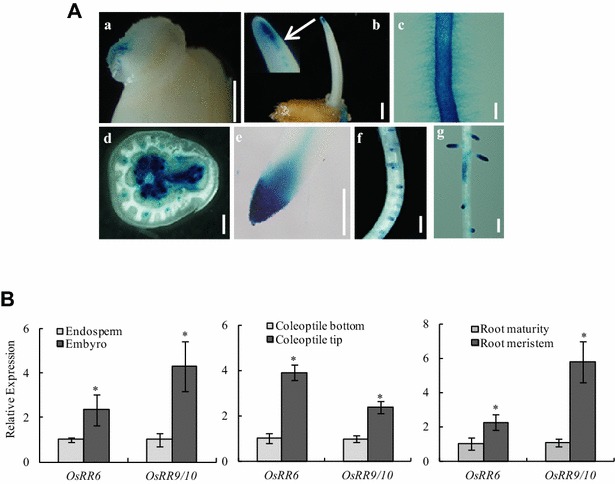
Histochemical localization of *TCSn::GUS* activity and gene expression in rice transgenic lines. Analysis was performed on transgenic lines germinated and grown hydroponically for 14 days. **A** (a) A 2-day-old germinated seed; (b–c) coleoptile (b) and primary root with root hair (c) in a 4-day-old germinated seed; (d–g) transverse section of stembase with axillary bud (d), primary root apical meristem (e), lateral root primordium (f), and primary seedling root with lateral root (g) in a 14-day-old seedling. Bars = 1 mm. **B** Expression of two Type-A regulator genes (*OsRR6* and *OsRR9/10*) in rice in embryo and endosperm, at the tip and bottom of the coleoptile and in the meristem and maturity zones of the root

To determine whether *TCSn* functions in monocotyledon rice and clarify the functions of cytokinins, we constructed a transgenic rice plant harboring beta-glucuronidase (GUS) driven by the synthetic promoter *TCSn* (Additional file 5; [22]). Cytokinins can stimulate cell division [40, 41]; therefore, we first analyzed the expression pattern of *TCSn::GUS* in rice seedlings, especially in the meristem. Strong GUS expression was detected in 2-day-old germinated seeds, indicating vigorous cell division (Fig. 2A[a]). Over time, GUS expression was found at the top of the coleoptile and was stronger in the primary root with root hair in 4d-old germinated seeds. As expected, GUS was expressed at the tip of the primary root and in the primordia of the lateral roots of 2-week-old rice seedlings (Fig. 2A[e–g]). The transverse section of the stembase with axillary buds also exhibited stronger GUS activity due to the high cell division capacity. Based on the histochemical localization of *TCSn::GUS* activity (Fig. 2A), the expression of two Type-A regulator genes (*OsRR6* and *OsRR9/10*) in rice was analyzed in the embryo and endosperm at the tip and bottom of the coleoptile and in the root meristem and maturity zones. *OsRR6* and *OsRR9/10* expression levels were higher in the embryo than in the endosperm, in the tip than the bottom of the coleoptile and in the meristem than the maturity of the root. These findings are in agreement with the changes in *TCSn::GUS* expression in tissues, suggesting that GUS activity is consistent with the functions of cytokinins [16, 40, 41] (Fig. 2B).

### *TCSn::GUS* activity in response to exogenous cytokinin

**Fig. 3:**
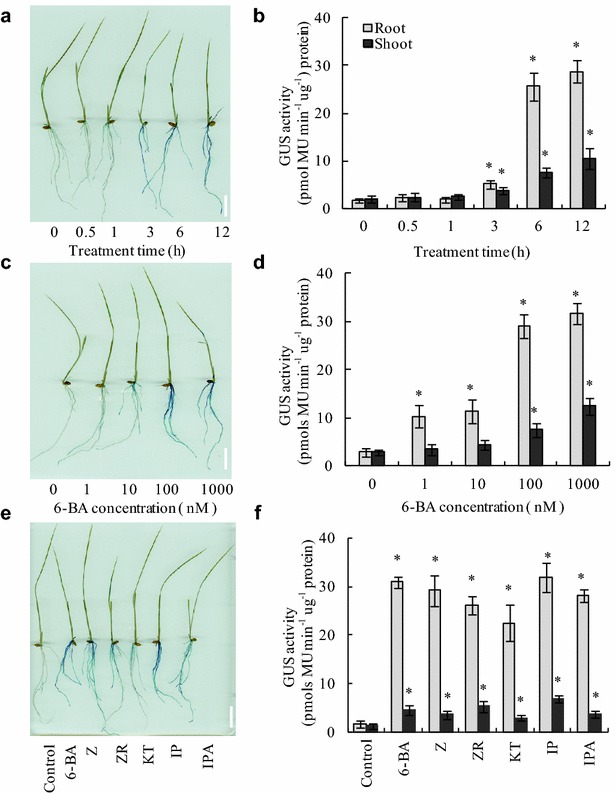
*TCSn::GUS* activity in response to exogenous cytokinins. Analysis was performed on transgenic lines germinated and grown hydroponically for 7 days followed by application of exogenous cytokinins. **a**, **b** Staining (**a**) and quantification (**b**) of GUS expression in response to application of 100 nM 6-benzylaminopurine (6-BA) for 12 h; **c**, **d** staining (**c**) and quantification (**d**) of *GUS* expression according to 6-BA concentration; **e**, **f** staining (**e**) and quantification (**f**) of GUS expression in response to treatment with 100 nM cytokinin fractions (Z, zeatin; ZR, zeatin riboside; KT, kinetin; iP, N6-(Δ2-isopentenyl) adenine; iPA, iso-pentenyl adenosine). Values are mean ± SD of four biological replicates. Bars = 2 cm. *P < 0.05 (ANOVA) between the control and the indicated treatments

To assess the sensitivity of *TCSn::GUS* to cytokinin, we grew transgenic rice seedlings hydroponically for 7 days and then treated them with exogenous cytokinin 6-BA (100 nM) for 12 h. GUS expression was notably induced in roots and shoots by 6-BA application at 3 h (Fig. 3a, b). Furthermore, the supply of 6-BA for 6 h increased GUS activity by 20-fold in roots and by sixfold in shoots compared with the controls. These data suggested that the histochemical staining of *TCSn::GUS* in roots was more pronounced than in shoots, similar to Arabidopsis [22]. Next, we analyzed GUS activity in response to application of 0–1000 nM 6-BA (Fig. 3c, d). Notably, only application of 1 nM 6-BA significantly enhanced GUS activity by fourfold in rice roots, whereas application of 10 nM 6-BA enhanced GUS activity twofold in rice shoots. Moreover, GUS activity in rice roots and shoots increased with increasing 6-BA concentrations. Finally, we evaluated the effect of different cytokinin fractions on GUS activity. The five cytokinin fractions had an effect similar to that of 6-BA (Fig. 3e, f). These findings suggest that *TCSn::GUS* expression in roots and shoots was increased by exogenous cytokinin application. Furthermore, we analyzed the expression of *OsRR6* and *OsRR9/10* (Additional file 6) in the root and found that expression of both matched the *TCSn::GUS* expression (Fig. 3), confirming that GUS activity is consistent with two marker genes expression for the cytokinin responses. Importantly, no reduction in GUS levels was observed after three generations of selfing of transgenic plants (Additional file 7), indicating that expression of *TCSn::GUS* is not subject to transgene silencing.

### Specificity of *TCSn::GUS* in response to exogenous cytokinin

**Fig. 4:**
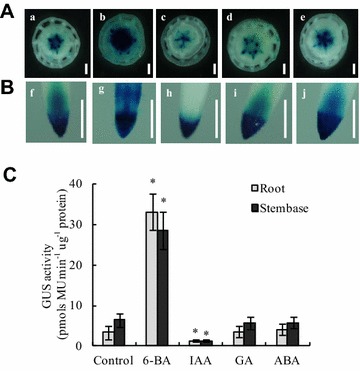
*TCSn::GUS* activity in response to exogenous phytohormones. Analysis was performed on transgenic lines germinated and grown hydroponically for 7 days followed by 3 h treatment with 6-benzylaminopurine (6-BA, 100 nM), indole-3-acetic acid (IAA, 1 μM), gibberellic acid (GA, 10 μM), and abscisic acid (ABA, 10 μM). **A**, **B** GUS expression at the stembase (**A**) and primary root tip (**B**); **C** quantification of GUS expression. Values are mean ± SD of four biological replicates. Bar = 0.5 mm. *P < 0.05 (ANOVA) between the control and the indicated treatments

To determine whether the *TCSn* synthetic promoter responds specifically to cytokinin, we investigated the change in *TCSn*:*:GUS* expression in response to three other phytohormones. As expected, GUS expression at the stembase and at the tip of primary root was similar to that of other *OsRR* genes. IAA treatment decreased GUS expression at the stembase and at the tip of the primary root in rice plants (Fig. 4c, h), which is consistent with the antagonism between auxin and cytokinin [42, 43]. Compared with the control, no GUS expression was observed when GA or ABA was applied (Fig. 4). Therefore, cytokinins specifically activate the synthetic promoter *TCSn*, whereas auxin, gibberellic acid, and abscisic acid do not.

## Conclusion

In this study, we analyzed the DNA-binding domains 5′-(A/G)GAT(T/C)-3′ in type-A OsRR family members and examined the relative expression of type-A *OsRR* genes in response to treatment with phytohormones. The strong version of *TCSn::GUS* was fused into rice plants by genetic transformation. We evaluated GUS expression in transgenic rice seedlings, including differences between generations and responses to exogenous application of cytokinin fractions and three phytohormones. A cytokinin-responsive type-A OsRR-binding element was clustered in the upstream sequences of 15 type-A *OsRR* genes. *TCSn::GUS* was expressed in the meristem of germinated rice seeds and seedlings. Furthermore, *TCSn::GUS* expression was induced specifically by exogenous application of cytokinin in rice seedlings. We report here a robust and sensitive synthetic sensor for monitoring the transcriptional output of the cytokinin signaling network in rice.

## Methods

### Construction of reporter vectors and transformation

The *TCSn* promoter sequence [22] was commercially synthesized by the Genewiz Company (Additional file 1). To construct the *TCSn::GUS* vector, we inserted the synthetic *TCSn* promoter sequence into the *Sal*I and *BamH*I sites of the pDR5::GUS vector. The *pDR5::GUS* construct was kindly provided by the Ping Wu laboratory at Zhejiang University, Hangzhou, China. The construct was transformed into callus initiated from rice seeds (cv. Shiokari) by *Agrobacterium tumefaciens* (strain EHA105)-mediated transformation. Rice embryonic calli were induced on N_6_ media, and transformation was performed by *Agrobacterium*-mediated cocultivation [44].

### Plant growth conditions and treatments

Transgenic rice seeds were surface-sterilized with 10% (v/v) H_2_O_2_ for 30 min and rinsed thoroughly with deionized water. The sterilized seeds were germinated on a plastic support netting (mesh, 1 mm^2^) mounted in plastic containers for 1 week. Uniform seedlings were selected and then transferred to a tank containing 7 L of International Rice Research Institute (IRRI) nutrient solution for 2 weeks at pH 5.5. IRRI nutrient solution (1.25 mM NH_4_NO_3_, 0.3 mM KH_2_PO_4_, 0.35 mM K_2_SO_4_, 1 mM CaCl_2_·2H_2_O, 1 mM MgSO_4_·7H_2_O, 0.5 mM Na_2_SiO_3_, 20 μM NaFeEDTA, 20 μM H_3_BO_3_, 9 μM MnCl_2_·4H_2_O, 0.32 μM CuSO_4_·5H_2_O, 0.77 μM ZnSO_4_·7H_2_O and 0.39 μM Na_2_MoO_4_·2H_2_O; pH 5.5) was supplied and replaced every 2 days. Plants were grown in a growth chamber at 30 °C during the day and 22 °C during the night with a 16-h light/8 h dark regime. The relative humidity was controlled at ~ 70%.

The cytokinin fractions (6-benzylaminopurine, 6-BA; zeatin, Z; zeatin riboside, ZR; kinetin, KT; N6-(Δ2-isopentenyl) adenine, iP; iso-pentenyl adenosine, iPA), 1 µM indole-3-acetic acid (IAA), 10 µM gibberellic acid (GA), and 10 µM abscisic acid (ABA) were added to the hydroponic media. The seedlings were treated with 100 nM 6-benzylaminopurine (6-BA) at 0, 0.5, 1, 3, and 6 h and with 0, 1, 10, 100, and 1000 nM 6-BA for 6 h.

### Quantitative reverse-transcription polymerase chain reaction analysis of gene expression

Total RNA was isolated from the roots of rice seedlings. RNA extraction, reverse transcription, and quantitative reverse-transcription polymerase chain reaction (qRT-PCR) were performed as described previously [45]. Primer sets for type-A *OsRRs* are listed in Additional file 8.

### Histochemical GUS staining and quantitative measurement of GUS activity

Histochemical GUS staining was performed as described previously [46]. The tissues were treated for 1 h with 1.8 M KOH solution heated to 90 °C and then treated with 1% HCl (v/v) solution for 5 min [47]. The stained tissues were photographed using an Olympus SZX2ILLK stereomicroscope equipped with a color charge-coupled device (CCD) camera (Olympus) [48].

For enzymatic GUS assays, tissues were ground in liquid nitrogen and transferred to microtubes containing 1 mL of the extraction buffer (50 mM sodium phosphate, pH 7.5; 10 mM Na_2_EDTA; 10 mM 2-mercaptoethanol; 0.1% Triton X-100; and 0.1% (w/v) sodium lauryl-sarco-sine) for total protein extraction. Beta-glucuronidase activity was measured fluorimetrically using 1 µg of total protein extract, as described previously [47].

### Data analysis

Experimental data were pooled for calculation of means and standard deviations (SDs) and subjected to one-way analysis of variance (ANOVA), followed by least significant difference (LSD) testing to determine the significance of differences between individual treatments. Data in Additional file 3 were pooled and analyzed by nonparametric tests, followed by two independent-samples *t* tests to determine the significance of differences between two independent samples. All statistical procedures were performed using SPSS version 11.0 (SPSS, Chicago, IL, USA).

## Additional files

**Additional file 1.** Amino acid alignment of the Myb-like domains from type-B response regulators.

**Additional file 2.** Gene sequences used for the bioinformatic analysis.

**Additional file 3.** Summary of 5′-(A/G)GAT(T/C)-3′ motifs in rice cytokinin type-A genes, control genes and random sequence.

**Additional file 4.** Relative transcript levels of type-A *OsRR* family genes revealed by qRT-PCR in root and shoot.

**Additional file 5.** Sequence of synthetic promoter *TCSn*.

**Additional file 6.** Relative expression of two type-A genes in rice.

**Additional file 7.** *TCSn::GUS* activity in T1 and T3 generations.

**Additional file 8.** The primers for qRT-PCR of type-A *OsRR* genes.
