# Supplementary material for: A sensitive synthetic reporter for visualizing cytokinin signaling output in rice
Source: Plant Methods. 2017 Oct 27;13:89. doi: 10.1186/s13007-017-0232-0 (PMC5658958; doi:10.1186/s13007-017-0232-0)
Supplement: Supplementary file 1 — Additional file 1. Amino acid alignment of the Myb-like domains from type-B response regulators. [file 13007_2017_232_MOESM1_ESM.docx]

**Additional file 1** Amino acid alignment of the Myb-like domains from type-B response regulators. Representative sequences from important Arabidopsis and rice type-B response regulators were aligned by DNAMAN. Residues above the alignment are majority in the Myb-like motif.
