# Supplementary material for: A sensitive synthetic reporter for visualizing cytokinin signaling output in rice
Source: Plant Methods. 2017 Oct 27;13:89. doi: 10.1186/s13007-017-0232-0 (PMC5658958; doi:10.1186/s13007-017-0232-0)
Supplement: Supplementary file 4 — Additional file 4. Relative transcript levels of type-A OsRR family genes revealed by qRT-PCR in root and shoot. [file 13007_2017_232_MOESM4_ESM.docx]

**Additional file 4** Relative transcript levels of type-A *OsRR* family genes revealed by qRT-PCR in root and shoot, *OsActin* as internal control. The wild type seedling germinated and grew hydroponically for 14 d. Values are means ± SD of four biological replicates.
