# Supplementary material for: A sensitive synthetic reporter for visualizing cytokinin signaling output in rice
Source: Plant Methods. 2017 Oct 27;13:89. doi: 10.1186/s13007-017-0232-0 (PMC5658958; doi:10.1186/s13007-017-0232-0)
Supplement: Supplementary file 5 — Additional file 5. Sequence of synthetic promoter TCSn. [file 13007_2017_232_MOESM5_ESM.docx]

**Additional file 5**

A concatemer of 24 repeats of the 5'-(A/G)GAT(C/T)T-3' binding motif

(2 repeats of 12 sites with random order), 270 bp

| 1 |  | C | A | A | A | **G** | **A** | **T** | C | T | T | T |
| --- | --- | --- | --- | --- | --- | --- | --- | --- | --- | --- | --- | --- |
| 2 |  | G | A | G | A | **G** | **A** | **T** | C | T | T | T |
| 3 |  | C | A | A | A | **G** | **A** | **T** | C | T | T | T |
| 4 |  | A | A | A | A | **G** | **A** | **T** | T | T | T | G |
| 5 |  | A | A | A | A | **G** | **A** | **T** | T | T | A | T |
| 6 |  | C | A | A | A | **G** | **A** | **T** | T | T | T | T |
| 7 |  | C | A | A | A | **G** | **A** | **T** | T | T | T | G |
| 8 |  | T | A | A | A | **G** | **A** | **T** | T | T | T | G |
| 9 |  | T | A | A | A | **G** | **A** | **T** | T | T | T | G |
| 10 |  | A | A | A | G | **G** | **A** | **T** | T | T | T | G |
| 11 |  | G | T | T | G | **G** | **A** | **T** | T | T | T | G |
| 12 |  | A | T | G | G | **G** | **A** | **T** | C | T | T | G |
|  |  |  |  |  |  |  |  |  |  |  |  |  |
|  |  |  |  |  |  |  |  |  |  |  |  |  |

Sequence of *TCSn*

AGTCAAAGATCTTTAAAAGATTTTGAAAGATCTCTCCAAAATCCTTTCAAAGATCTTTAAAAGATTTATAAAAATCTTTGCAAAATCCAACCAAAGATTTTGTAAAGATTTTGCAAGATCCGATCAAAATCTTTAGCTAGTCAAAGATCTTTAAAAGATTTTGAAAGATCTCTCCAAAATCCTTTCAAAGATCTTTAAAAGATTTATAAAAATCTTTGCAAAATCCAACCAAAGATTTTGTAAAGATTTTGCAAGATCCGATCAAAATCTTTAGCTAGCCAAGACCCTTCCTCTATATAAGGAAGTTCATTTCATTTGGAGAGGATCTGTATTTTTACAACAATTACCAA
