# Supplementary material for: A sensitive synthetic reporter for visualizing cytokinin signaling output in rice
Source: Plant Methods. 2017 Oct 27;13:89. doi: 10.1186/s13007-017-0232-0 (PMC5658958; doi:10.1186/s13007-017-0232-0)
Supplement: Supplementary file 6 — Additional file 6. Relative expression of two type-A genes in rice. [file 13007_2017_232_MOESM6_ESM.docx]

**Additional file 6** Expression pattern of *OsRR6* and *OsRR9/10* in response to exogenous cytokinin supplies. Analysis was performed on wild type seedling germinated and grew hydroponically for 7 d followed by application of exogenous cytokinins. (a) in response to application of 100 nM 6-benzylaminopurine (6-BA) for 6 h; (b) in response to 6-BA concentration; (c) in response to treatment with 100 nM cytokinin fractions (Z, zeatin; ZR, zeatin riboside; KT, kinetin; iP, N6-(Δ2-isopentenyl) adenine; iPA, iso-pentenyl adenosine). Values are means ± SD of four biological replicates.
