# Supplementary material for: A sensitive synthetic reporter for visualizing cytokinin signaling output in rice
Source: Plant Methods. 2017 Oct 27;13:89. doi: 10.1186/s13007-017-0232-0 (PMC5658958; doi:10.1186/s13007-017-0232-0)
Supplement: Supplementary file 7 — Additional file 7. TCSn::GUS activity in T1 and T3 generations. [file 13007_2017_232_MOESM7_ESM.docx]

B

A

**Additional file 7** *TCSn::GUS* activity in T1 and T3 generations. Analysis was performed on transgenic lines germinated and grew hydroponically for 2 d. (A) Photo of GUS expression. (B) Quantification of GUS expression in roots and shoots. Bar = 0.5 mm. Values are means ± SD of four biological replicates.
