# Supplementary material for: A sensitive synthetic reporter for visualizing cytokinin signaling output in rice
Source: Plant Methods. 2017 Oct 27;13:89. doi: 10.1186/s13007-017-0232-0 (PMC5658958; doi:10.1186/s13007-017-0232-0)
Supplement: Supplementary file 8 — Additional file 8. The primers for qRT-PCR of type-A OsRR genes. [file 13007_2017_232_MOESM8_ESM.docx]

**Additional file 8**

The primers for qRT-PCR of type-A Os*RR* genes

| Gene | Locus ID | Primer sequence |
| --- | --- | --- |
| *OsRR1* | LOC_Os04g36070 | 5'-AGGATCAGCAGATGCATGAATG-3'  5'-GAGACGCTGTACGTCCTTGCTT-3' |
| *OsRR2* | LOC_Os02g35180 | 5'-ACGATCTTCTCAAAGCCATCAAG-3'  5'-TGAGAGGCTTAAGGATGAAATCCT-3' |
| *OsRR3* | LOC_Os02g58350 | 5'-GAGGGGGCTGAAGACTTTTT-3'  5'-CTGCTACAACGCAGCATCTC-3' |
| *OsRR4* | LOC_Os01g72330 | 5'-GAAGCCAAGATTGTCCTCCA-3'  5'-CAGAAAAAGAGGCCAAAATCA-3' |
| *OsRR5* | LOC_Os04g44280 | 5'-ACCGAATGTGAGCATGATTATCA-3'  5'-CCTTGACCTTCTTCAGGAGTTCATA-3' |
| *OsRR6* | LOC_Os04g57720 | 5'-CCGAGGACTTCCTGCTCA'  5'-TCATCCTCTCCATGATCCAA-3' |
| *OsRR7* | LOC_Os07g26720 | 5'-TGCTCAAGAAGATCAAGGAATCG-3'  5'-GGCACGTTCTCTGACGACATTAT-3' |
| *OsRR8* | LOC_Os08g28950 | 5'-GGATGGAGGAGCAAAGGATT-3'  5'-GAGCAAACAAAGCACAAAATAGC-3' |
| *OsRR9/10* | LOC_Os11g04720  LOC_Os12g04500 | 5'-TCATGAGGACAGCCCAATTTCTA-3'  5'-TGCAGTAGTCTGTGATGATCAGGTT-3' |
| *OsRR11* | LOC_Os02g42060 | 5'-CTAGGCTCGGAACCAAATG-3'  5'-ACGGGGATCTTCTTCAGCTT-3' |
| *OsRR12* | LOC_Os08g26990 | 5'-CTTGGATGGAGGAGCAAAGG-3'  5'-CCATCTTGATCATTGTTTC-3' |
| *OsRR13* | LOC_Os04g13480 | 5'-GCTTCGATGGAGGAGCAA-3'  5'-TCTTTCTTCATCTTGCCAATCA-3' |
| *OsActin* | LOC_Os03g50890 | 5'-CAACACCCCTGCTATGTACG-3  5'-CATCACCAGAGTCCAACACAA-3' |
